# Genetic voltage indicators

**DOI:** 10.1186/s12915-019-0682-0

**Published:** 2019-09-12

**Authors:** Yuki Bando, Christiane Grimm, Victor H Cornejo, Rafael Yuste

**Affiliations:** 1 Neurotechnology Center, Department Biological Sciences, Columbia University, 550 W 120th Street, New York, NY 10027 USA; 2 Present address: Department Organ and Tissue Anatomy, Hamamatsu University School of Medicine, Hamamatsu, Shizuoka, 431-3192 Japan

## Abstract

As a “holy grail” of neuroscience, optical imaging of membrane potential could enable high resolution measurements of spiking and synaptic activity in neuronal populations. This has been partly achieved using organic voltage-sensitive dyes in vitro, or in invertebrate preparations yet unspecific staining has prevented single-cell resolution measurements from mammalian preparations in vivo. The development of genetically encoded voltage indicators (GEVIs) and chemogenetic sensors has enabled targeting voltage indicators to plasma membranes and selective neuronal populations. Here, we review recent advances in the design and use of genetic voltage indicators and discuss advantages and disadvantages of three classes of them. Although genetic voltage indicators could revolutionize neuroscience, there are still significant challenges, particularly two-photon performance. To overcome them may require cross-disciplinary collaborations, team effort, and sustained support by large-scale research initiatives.

*“...And supposing there were a machine, so constructed as to think, feel, and have perception, it might be conceived as increased in size, while keeping the same proportions, so that one might go into it as into a mill.” (Leibniz, Monadologie, 1714*).

## Introduction

Like Leibniz walking into his mill of the mind, imagine watching, in real time, the workings of the nervous system, with neurons receiving excitatory and inhibitory postsynaptic potentials (EPSPs and IPSPs, respectively), integrating them into a common electrical response, and generating action potentials (APs) that are transmitted to other neurons. Such a dream experiment, in a way a “holy grail” of neuroscience, could be carried out by imaging membrane potential. A flavor of this can already be appreciated from calcium imaging [1–3], where, using either organic or genetically encoded calcium indicators, one can monitor the activity of neuronal populations in awake behaving animals, albeit at slow time resolution and without the ability to observe individual spikes during high frequency spike trains or to measure synaptic potentials [4–6].

Voltage imaging of neurons is difficult for many reasons. Although the membrane potential is quite significant in amplitude (up to a tenth of a volt), it exists in a confined fraction of space, the thin plasma membrane and its associated Debye length, only a few nanometers thick. Because of this, to measure the electric field, sensors need to be targeted with nanometer precision, with little room for error. Moreover, sensors have to be specifically targeted to the plasma membrane, since the vast majority of cellular membranes are intracellular, which, when labelled with voltage sensors, only contribute background to the signal. On top of this targeting challenge, the sheer thinness of the membrane means that only few sensor molecules can be positioned there, so voltage changes can only be reported using very few photons, demanding efficient chromophores, strong light sources, and temporal or spatial averaging. However, membrane voltage signals are millisecond fast and neurons have rich dendritic or axonal morphologies where voltage signals need to be measured, rendering spatial or temporal averaging problematic. To complicate things further, even if targeting was efficient and labeled all cells and processes, the tangle of the mammalian neuropil remains optically unresolvable to conventional microscopy. Also, membrane potentials are graded in amplitude, so measurements need to have a significant dynamic range with, ideally, linear transfer functions in the physiological range of − 100 to 100 mV. A final difficulty arises as the plasma membrane is not just another cellular compartment, but precisely the one that protects the neuron from the outside, and whose integrity is of paramount importance. This makes it extremely sensitive to any perturbation, from adding additional molecules or charges that can interfere with its biochemical or electrical properties, to photodamage from the generation of oxygen free radicals due to the photoexcitation of voltage indicators or of endogenous chromophores.

This forbidding set of difficulties has not stopped researchers from tackling voltage imaging [7–9], resulting in a variety of different methodological approaches demonstrating great ingenuity [10]. Indeed, methods to optically measure membrane potential have exploited strategies as diverse as (i) repartitioning, where chromophores move in and out of the membrane with voltage changes; (ii) reorientation, where the electric field changes the relative alignment of the chromophore with respect to the membrane; (iii) electrochromism, where the membrane potential modulates the ground and excited states of the chromophore, altering the excitation or emission wavelength; (iv) Förster resonance energy transfer (FRET), where voltage-induced conformational or spectral changes alter the efficiency of energy transfer of chromophores; (v) quenching, where the membrane potential affects the molecular interactions that decrease the fluorescence’s intensity; (vi) voltage-induced dimerization/aggregation of chromophores, altering their spectra; (vii) electro-optic modulation of the second harmonic generation (SHG) of chromophores; (viii) plasmonic effect of nanoparticles to amplify signals from nearby chromophores; and (ix) imaging refractive index or other intrinsic optical changes in the cell due to its electrical activity.

Exploiting some of these mechanisms, in the past four decades researchers have synthetized organic voltage-sensitive dyes to measure membrane potential in vitro and in vivo [7–9, 11–14]. These dyes have been particularly effective in invertebrate preparations with large and robust neurons and with little neuropil [15–17], and also in some mammalian preparations, either in vitro [18, 19], or by injecting dyes into individual cells [20, 21], or using them for bulk tissue measurements in vitro [22, 23] or in vivo, but without single cell resolution [8]. In spite of this pioneering work, voltage imaging of mammalian preparations in vivo with single cell resolution has remained a challenge, and imaging of neural circuits activity in vivo is instead generally done with calcium indicators, combining it with two-photon excitation for optical penetration and sectioning [4, 24, 25].

**Fig. 1:**
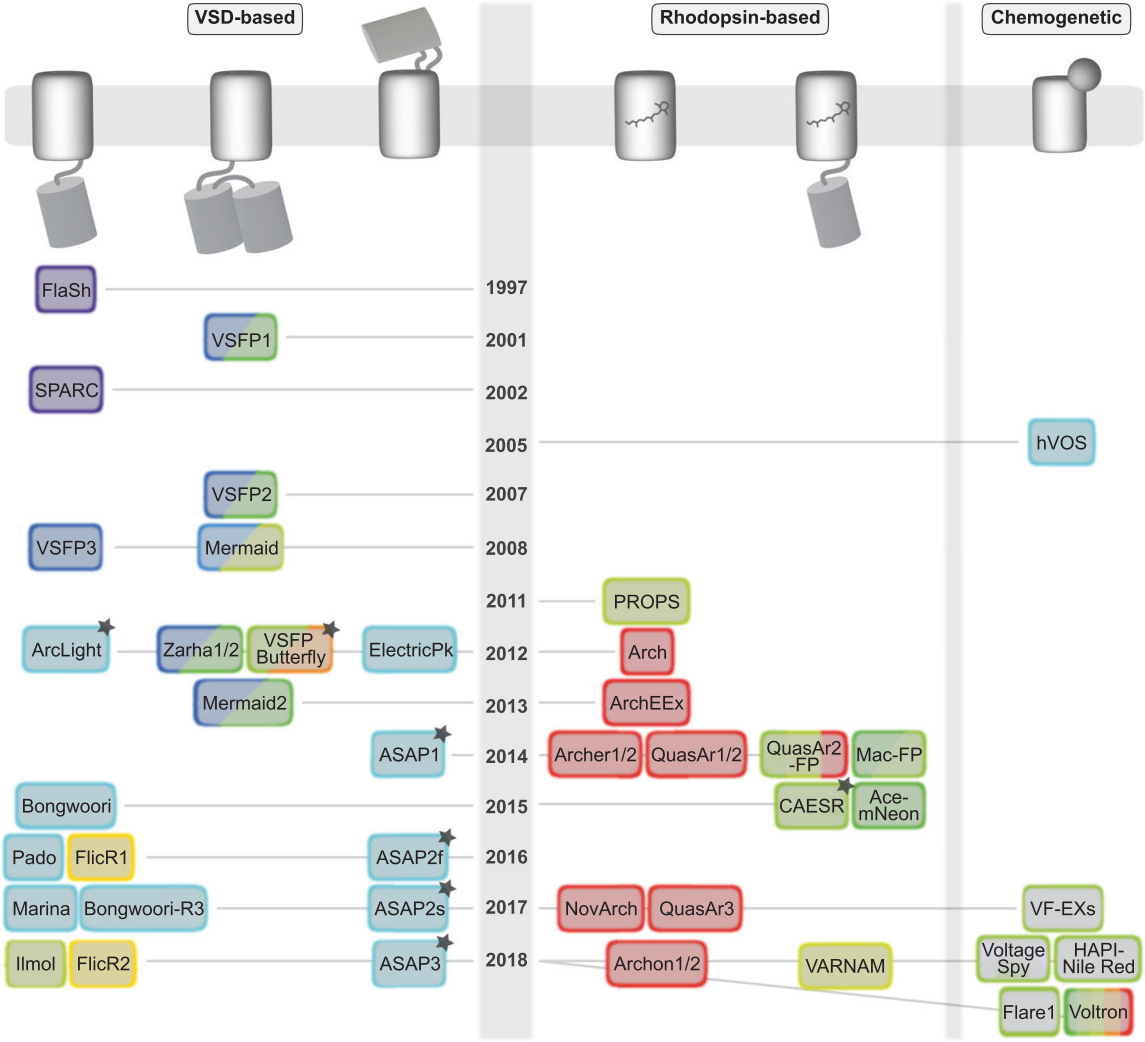
Historical overview of genetic voltage indicators. Sensors fall into three distinct families based on voltage sensing domains (VSD; *left*), microbial rhodopsins (*middle*), or chemogenetic probes (*right*) and are arranged chronologically according to year of first report. Color of the box refers to the activation wavelength reported in the paper or inferred from the spectrum of the fluorescent protein. *Black stars* denote reported two-photon measurements. Note that HAPI-Nile Red and Voltron are also rhodopsin-based. See text for references

The recent development of genetically encoded voltage indicators (GEVIs) represents a new strategy that, by using protein engineering, could overcome some of the limitations of the organic voltage-sensitive dyes (Fig. 1). Building on the successful development of genetically encoded calcium indicators [26], the discovery of a voltage-sensitive domain (VSD) from a phosphatase [27, 28] has enabled building a family of GEVIs by coupling it to fluorescent proteins in different configurations (Fig. 1, left). In addition, a second family of GEVIs has been developed based on microbial rhodopsins, which show weak, yet voltage-sensitive, fluorescence [29]. Finally, a third category of genetic voltage sensors exploit a hybrid approach, with interacting organic and protein components [30], harnessing the joint benefits of chemical and genetic designs. In the following sections we provide a brief review of these three families of genetic voltage indicators, and provide a comparison of their performance in Table 1. Given how quickly this field is progressing, our review is just a snapshot in time and we encourage the reader to keep abreast of new voltage indicators as they are published.

**Table 1:** Comparative performance of genetically targeted voltage indicators. Values extracted from the literature. *NR* not reported, *RT* room temperature

	λ_ex_ [nm]	λ_em_ [nm]	Rise time (depol.) [ms]	Decay time (hyperpol.) [ms]	Intensity ~[W/cm^2^]	SNR	Res. spike rate [Hz]	ΔF/F for 100 mV	Bleaching	Reference
*VSD-based*										
ArcLight Q239	480	520	τ_1_ = 9 (50%) τ_2_ = 48 (RT)	τ_1_ = 17 (79%) τ_2_ = 63 (RT)	Xenon arc lamp	3.7 per AP	10	−35%	τ = 244 s	Jin 2012 [34]; Bando 2019 [49]
ArcLight-MT	480	535	τ = 84.8 (RT)	τ = 91.9 (RT)	Mercury arc lamp	5.6 per AP	10	−20%	τ = 360 s	Kwon 2017 [46]; Bando 2019 [49]
ASAP1	472	525	τ_1_ = 2.1 (60%) τ_2_ = 71.5 (RT)	τ_1_ = 2.0 (43.7%) τ_2_ = 50.8 (RT)	10^− 1^	14.6 per AP	200	− 20%	τ = 35 m	St-Pierre 2014 [38]
ASAP2f	470	525	τ_1_ = 2.8 (81%) τ_2_ = 135 (RT)	τ_1_ = 2.4 (71%) τ_2_ = 155 (RT)	10^0^	5 per AP	100	−20%	τ = 404 s	Yang 2016 [39]; Chamberland 2017 [41]
ASAP2s	480	525	τ_1_ = 5.2 (56%) τ_2_ = 63 (RT)	τ_1_ = 24 (49%) τ_2_ = 106 (RT)	10^0^	8 per AP	100	−38%	τ_1 =_ 121 s (69%) τ_2 =_ 1017 s	Chamberland 2017 [41]
ASAP3	484	525	τ_1_ = 3.7 (81%) τ_2_ = 48 (RT)	τ_1_ = 16 (81%) τ_2_ = 102 (RT)	10^−1^	2.6 per AP (2P)	100	−50%	19.6%/first 10 s 0.99%/m after that (2P)	Chavarha 2018 [40]
Bongwoori	472	496	τ_1_ = 8 (91%) τ_2_ = 30 (RT)	τ = 7 (RT)	Xenon arc lamp	19 per AP	60	−15%	> 450 s	Piao 2015 [37]; Lee 2017 [91]
Bongwoori-R3	472	497	τ_1_ = 7 (90%) τ_2_ = 45 (RT)	τ_1_ = 6 (91%) τ_2_ = 46 (RT)	Xenon arc lamp	52 per AP	65	−20%	> 450 s	Lee 2017 [91]
FlicR1	561	595	τ_1_ = 3 (90%) τ_2_ = 42 (RT)	τ_1_ = 2.8 (70%) τ_2_ = 18 (RT)	10^1^	6 per AP	100	6.40%	τ = 150 s	Abdelfattah 2016 [42]; Kannan 2018 [44]
FlicR2	561	630	τ_1_ = 2.9 τ_2_ = 29.5 (RT)	τ_1_ = 3.1 τ_2_ = 28.5 (RT)	10^0^	NR	NR	12.90%	NR	Kannan 2018 [44]
Marina	488	520	τ = 29.2 (RT)	τ_1_ = 15.6 (61%) τ_2_ = 59.4 (RT)	10^0^	4.5 per AP	NR	29.20%	τ = 206 s	Platisa 2017 [43]
Mermaid	455	480 (donor) 575 (acceptor)	τ_1_~ 12 τ_2_~200 (RT)	τ~ 128 (RT)	10^0^	NR	100	~ 30% **Δ**R/R	NR	Tsutsui 2008 [28]
VSFP Butterfly1.2	483	542 (donor) 594 (acceptor)	τ_1_~1.5 (35%) τ_2_~15 (RT)	NR	Xenon arc lamp	NR	40	5% **Δ**R/R	NR	Akemann 2012 [92]
*Rhodopsin-based*										
Arch	640	687	< 1	< 1	10^3^	NR	NR	40% at 640 nm	NR	Kralj 2012 [29]; Maclaurin 2013 [58]; Hochbaum 2014 [93]
Arch (D95N)	640	687	τ_1_ = 0.5 (20%) τ_2_ = 41 (RT)	NR	10^3^	NR	NR	50% at 640 nm	NR	Kralj 2012 [29]; Maclaurin 2013 [58]
Archer1	655	NR	Like Arch	Like Arch	10^2^	NR	40	85% at 655 nm	NR	Flytzanis 2014 [53]
QuasAr1	640	715	τ_1_ = 0.05 (94%) τ_2_ = 3.2 (RT)	Similar to rising	10^2^	20–30 per AP	NR	32% at 640 nm	τ = 440 s	Hochbaum 2014 [93]
QuasAr2	640	715	τ_1_ = 1.2 (68%) τ_2_ = 11.8 (RT)	Similar to rising	10^2^	40–70 per AP	NR	90% at 640 nm	τ = 1020 s	Hochbaum 2014 [93]
QuasAr3	Like QuasAr2	Like QuasAr2	τ_1_ = 1.2 (77%) τ_2_ = 10.0 (34 °C)	τ_1_ = 0.9 (91%) τ_2_ = 9.0 (34 °C)	10^2^	27 per AP	NR	54% at 640 nm	NR	Adam 2018 [55]
Archon1	637	NR	τ_1_ = 0.6 (88%) τ_2_ = 8.1 (34 °C)	τ_1_ = 1.1 (88%) τ_2_ = 13 (34 °C)	10^1^–10^2^	21 per AP	NR	43% at 637 nm	0.01%/s	Piatkevich 2018 [57]
Archon2	637	NR	τ_1_ = 0.6 (70%) τ_2_ = 6.7 (34 °C)	τ_1_ = 0.17 (92%) τ_2_ = 7.0 (34 °C)	10^1^–10^2^	16 per AP	200	19% at 637 nm	0.03%/s	Piatkevich 2018 [57]
QuasAr2-mOrange	549	565	τ_1_ = 3.9 (60%) τ_2_ = 27 (23 °C)	τ_1_ = 4.3 (45%) τ_2_ = 26 (23 °C)	10^1^	9 per AP	NR	−10%	NR	Zou 2014 [61]
MacQ-mCitrine	515	530	τ_1_ = 2.8 (74%) τ_2_ = 71 (RT)	τ_1_ = 5.4 (77%) τ_2_ = 67 (RT)	10^1^	NR	NR	−20%	1.3%/s	Gong 2014 [60]; Gong 2015 [52]
Ace2-4aa-mNeon	505	515	τ_1_ = 0.37 (58%) τ_2_ = 5.5 (RT)	τ_1_ = 0.5 (60%) τ_2_ = 5.9 (RT)	10^1^	NR	NR	−12%	0.6%/s	Gong 2015 [52]
VARNAM	558	605	τ_1_ = 0.88 τ_2_ = 5.2 (RT)	τ_1_ = 0.80 τ_2_ = 4.7 (RT)	10^1^	36 per AP	100	−14% for 120 mV	τ = 256 s	Kannan 2018 [44]
*Chemogenetics based*										
hVOS	480	535	< 1	< 1	Mercury arc lamp	NR	667	34%	NR	Chanda et al. 2005 [70]
Flare1	488	570	τ_1_ = 0.92 (96%) τ_2_ = 23.5	τ_1_ = 1.41 (91%) τ_2_ = 25.6	10^0^	53	20	35.9%	NR	Xu et al. 2018 [74]
Voltron 525	532	553	τ_1_ = 0.64 (61%) τ_2_ = 4.1	τ_1_ = 0.78 (55%) τ_2_ = 3.9	10^0^	4.4	10	30%	τ = 206 s	Abdelfattah 2016 [42]
VF-EX	525	540	< 1	< 1	NR	20	3	21%	NR	Liu et al. 2017 [77]
VoltageSpy	525	540	< 1	< 1	10^−1^–10^0^	7.7	NR	60%	NR	Grenier et al. 2019 [82]
HAPI-Nile	540–552	581	τ_1_ = 1.9 (85%)	τ_1_ = 1.9 (85%)	10^1^	12.4	10	5.50%	NR	Sundukova et al. 2019 [83]

### Voltage-sensitive domain-based GEVIs

**Fig. 2:**
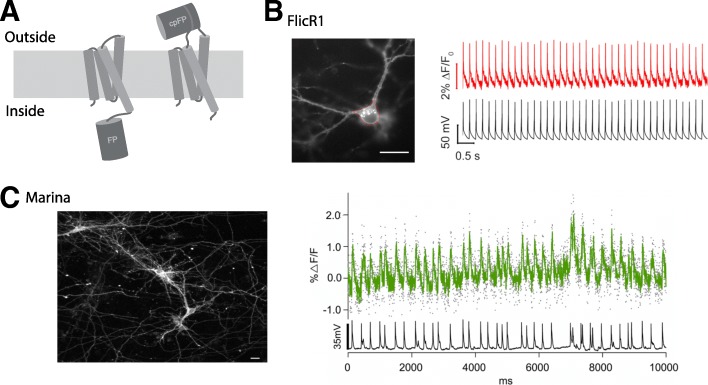
Recent VSD-based GEVIs. **a** Schematic drawing of two configurations of VSD-based GEVIs. *Left*: VSD fusion with intracellular fluorescent protein (FP). *Right*: VSD insertion with extracellular circularly permuted FP. **b** *Left*: Expression of FlicR1, a red-shifted indicator with flipped polarity, in dissociated hippocampal neuron. *Right*: optical (*red*) and electrical (*black*) responses to action potentials at 5 Hz, recorded with one-photon imaging. Modified with permission from [43]. **c** *Left*: Expression of Marina, a green indicator with flipped polarity in cultured hippocampal neurons. *Right*: Spontaneous spiking activity in a cortical neuron from an acute brain slice recorded with one-photon imaging. Modified from [44] with permission

VSD-based voltage indicators consist of a VSD and a fluorescent protein (Fig. 2a). The first VSD-based voltage indicator, FlaSh, used a VSD from a voltage-gated potassium channel [31] but was of limited use in mammalian preparations. More recently, the VSD of a phosphatase from *Ciona intestinalis* [27] has been systematically used to build GEVIs with improved membrane trafficking and enhanced performance [32, 33]. A screen of fluorescent proteins fused with this VSD resulted in ArcLight, composed of a VSD and a mutated super ecliptic pHluorin [34]. Although ArcLight had a good voltage sensitivity, its slow fluorescence kinetics result in low signal amplitude and limited temporal resolution for spike detection. To speed kinetics, mutations were introduced into the *Ciona* VSD, yielding improved ArcLight variants [35–37]. As an alternative to *Ciona*’s VSD, the VSD of another voltage-sensitive phosphatase from *Gallus gallus* was used to insert a circularly permuted superfolder GFP into the VSD’s extracellular loop, between the third and fourth transmembrane helices, to obtain faster voltage indicators, named accelerated sensor of action potentials (ASAP) [38–41]. More recently, efforts have been made to flip the polarity of optical signals; as opposed to some of the earlier indicators, these new voltage indicators (Marina, FlicR1, and FlicR2) increase in brightness when the membrane is depolarized and exhibit lower fluorescence at resting membrane potentials (Fig. 2b, c) [42, 43]. Also, red-shifted VSD-based GEVIs have recently been developed (Fig. 2b) [42, 44, 45].

VSD-based GEVIs have been used successfully for measurements of both single-neuron and neuronal circuits, enabling recording of membrane potential dynamics in small neuronal compartments, difficult to access with conventional electrophysiological methods. For example, in vitro measurements of the membrane potential in dendritic spines have been done with ArcLight, combining one-photon voltage imaging with two-photon glutamate uncaging [46]. Also, back-propagating action potentials in dendrites were recorded using ASAP2s with two-photon microscopy [40]. VSD-based GEVIs have also been used in vivo. With one- or two-photon wide-field voltage imaging, one can image sensory-evoked or spontaneous potentials from larger territories, albeit without single cell resolution [47–49]. Monitoring subthreshold membrane potential dynamics and action potentials with cellular resolution has been achieved in vivo using VSD-based GEVIs in *Drosophila* [39, 50]. But voltage imaging with single cell resolution in vivo has been challenging in mammalian preparations, due to light scattering and poor signal to noise ratio (SNR). Recently, both ArcLight-MT and the newly developed ASAP3 were used to report subthreshold potentials and spontaneous action potentials in awake or anesthetized mice in vivo under two-photon excitation with single cell resolution [49]. Also, voltage imaging and calcium imaging have also been recently combined in fruit flies in vivo [39].

Although the performance of VSD-based GEVIs has improved, voltage imaging using them is still challenging. Further advances appear necessary, especially for in vivo imaging. In particular, better performance under two-photon excitation and developing red-shifted indicators for multi-color imaging and combination with optogenetics would be desirable. It seems also important to engineer brighter VSD-based GEVIs to obtain higher SNRs, comparable with calcium imaging. Finally, like with other voltage indicators, fast photobleaching of VSD-based GEVIs can prevent long-term monitoring of membrane potential dynamics. To overcome photobleaching, improving Marina- and FlicR-type GEVI seems particularly promising, as they show low fluorescence during resting state, and become brighter when membrane potential is depolarized.

### Rhodopsin-based GEVIs

**Fig. 3:**
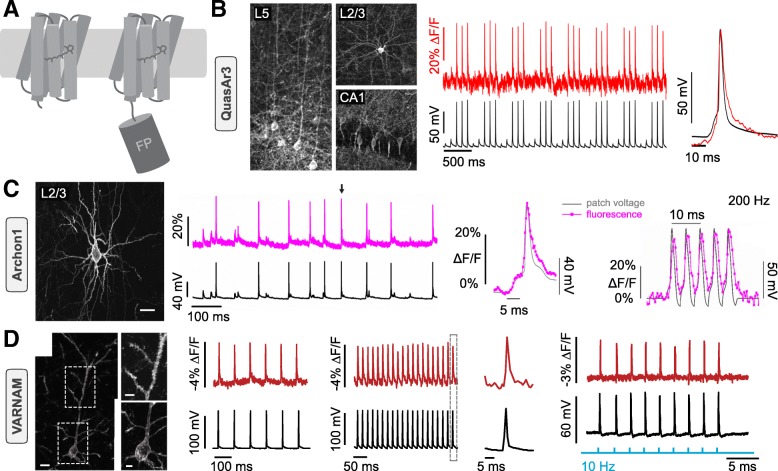
Recent rhodopsin-based GEVIs. **a** Representation of two kinds of rhodopsin-based GEVIs with PROPS type GEVI (*left*) and eFRET-based GEVI (*right*). **b** *Left*: Confocal images of QuasAr3 expression in brain slices; bar 50 μm. *Middle*: Patch-clamp recordings (*black*) with corresponding fluorescence traces (*red*) in acute brain slices. *Right*: overlay of electrical and optical signal for a single AP. Modified with permission from [55]. **c** *Left*: Expression of Archon1 in acute brain slices; bar 25 μm. *Middle*: Archon1 fluorescence (*pink*; single trial) and related electrical traces (*black*) in cultured cells with overlay of both signals for the AP indicated by the arrow. *Right*: Fluorescence changes (single trial) of Archon 1 following action potential-like voltage changes (*black*) of 200 Hz in a voltage-clamped neuron in culture. Modified with permission from [57]. **d** *Left*: Confocal image of VARNAM expression in pyramidal neurons in fixed postnatal brain slices. *Middle*: Concurrent optical (*red*) and electrical recordings (*black*) evoked by 10 Hz (*left*) and 50 Hz (*right*) current injections with overlay of both signals for indicated AP. *Right*: Changes in membrane potential driven by activation of the channelrhodopsin Cheriff (*blue*) monitored electrically (*black*) and optically (*red*). Modified with permission from [44]

GEVIs based on microbial rhodopsins fall into two distinct classes. One uses the rhodopsin both as voltage sensor and fluorescent reporter while the other uses a voltage-sensing rhodopsin linked to a fluorescent tag (Fig. 3a). The first microbial rhodopsin-based voltage sensor was PROPS (proteorhodopsin optical proton sensor) [51]. The authors found that, in green-absorbing proteorhodopsin, the protonation state of the retinal Schiff base (RSB), which covalently attaches the chromophore to the apoprotein, largely determines the color and fluorescence of the rhodopsin. They reasoned that a change in membrane voltage should influence the local electrochemical potential around the RSB and thereby alter the fluorescence of the protein [51]. Through mutagenesis, the natural light-activated ion transport activity of the microbial rhodopsin was abolished and the RSB pk_a_ was shifted to sense membrane potentials in a physiological range. The use of PROPS was limited to *Escherichia coli*, but, exploiting a similar sensing mechanism, Archaerhodopsin 3 of the haloarchaea *Halorubrum sodomense*, known as Arch, was subsequently developed for voltage imaging of mammalian neurons [29]. In recent years, improvements of rhodopsin-based sensors have mainly stemmed from mutations in Arch [52, 53], yielding improved indicators like QuasAr 1 to 3 [54, 55], NovArch [56], and, recently, Archon 1 and 2 [57] (Fig. 1). Both QuasAr3 and Archon1 have been used to successfully record action potential trains in vitro with good SNR [55, 57] (Table 1) and have been used in vivo, albeit with one photon excitation [55, 57].

The combination of sensor and reporter in one small protein in microbial rhodopsins seems elegant and enables response times in the sub-millisecond range [29, 51, 54, 58], and additionally, large sensitivities (as ΔF/F per 100 mV) of 30 to 90% [53–57] render them very promising. Nevertheless, as voltage indicators, microbial rhodopsins suffer from drawbacks that even the latest variants could not overcome. As proteins optimized for ion transport and not fluorescence, their quantum yield is usually orders of magnitude lower than that of fluorescent proteins like GFP [29], generating low brightness and demanding high illumination intensities in the range of several tens to hundreds of W/cm^2^, even for the latest variants [55, 57]. To improve brightness, microbial rhodopsins have been combined with fluorescent proteins, yielding the second subgroup of rhodopsin-based sensors: the electrochromic FRET (eFRET) GEVIs (Fig. 3a), where the rhodopsin serves essentially as a VSD. Here, a fluorescent protein is C-terminally fused to the seventh transmembrane helix, enabling voltage-sensitive non-radiative quenching of the fluorophore by the rhodopsin, a mechanism already explored earlier with organic dyes [59]. Initial approaches fused the Mac rhodopsin, a light-driven proton pump from *L. maculans* (absorption peak 550 nm) to mCitrine or mOrange2 [60]. Although slightly slower than the pure rhodopsin sensors, MacQ-mCitrine and mOrange2 still generated a full amplitude response within 5 ms and reliably reported action potentials in cultured neurons with 5–7% ΔF/F per spike [60]. Following the same approach, QuasAr2 was fused to several fluorescent proteins (eGFP, Citrine, mOrange2, mRuby2) yielding sensors with similar kinetics and sensitivities [61]. Using the faster *Acetabularia* rhodopsin (Ace) as a quencher for mNeonGreen, response times could be significantly accelerated without a loss in sensitivity [62]. The latest and most red-shifted addition to the eFRET GEVIs is the recently published VARNAM, which also uses Ace coupled to the fluorescent protein mRuby3. VARNAM requires low light intensities (1.5 W/cm2), retains the fast kinetics of Ace-mNeonGreen, and shows high photostability [44], while its red-shifted activation makes it readily combinable with blue-light activated optogenetic actuators. However, even VARNAM was not able to overcome a shortcoming of rhodopsin-based GEVIs: weak performance under two-photon illumination [44].

### Chemogenetic indicators

Although GEVIs have the advantage that they can be genetically targeted to plasma membranes and cellular populations, they can have shortcomings due to low brightness, poor photostability, and slow kinetics. But, as mentioned, optical measurements of cellular membrane potential have been performed for decades with small organic synthetic molecules [12, 13, 15]. These dyes are voltage-sensitive, often because of electrochromism, and can have large fractional changes in fluorescence and excellent kinetics response and photophysical properties [8, 11, 63]. At the same time, these small lipophylic molecules generate unspecific staining of tissue, severely compromising SNR and cellular delimitation. To circumvent these problems, a hybrid strategy has emerged, using chemical and genetic indicators together: combining the optical properties of small molecule fluorophores with genetic targeting (Fig. 1) [30, 64–66]. The term “chemogenetics”, normally used for a small molecule that activates genetically engineered proteins, has been applied to these hybrid voltage indicators [67]. We review three general classes of chemogenetics indicators, according to the molecular mechanism of the sensing domain and fluorescent reporter.

#### FRET-based chemogenetic sensors

**Fig. 4:**
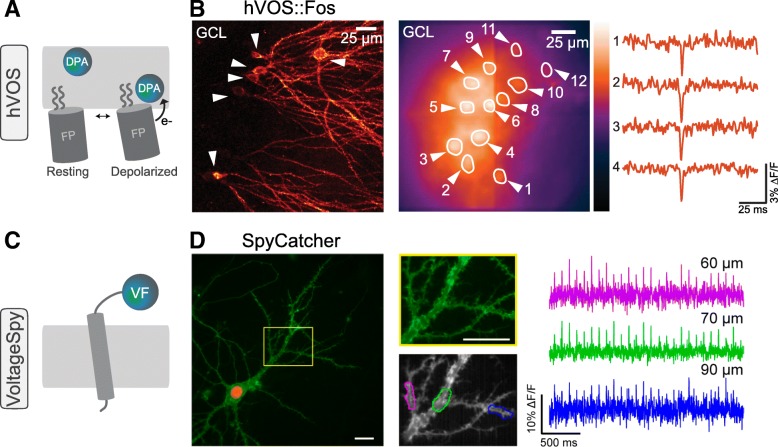
Chemogenetic voltage indicators. **a** Schematic representation of hVOS, consisting of a fluorescent protein anchored to the plasma membrane, combined with a non-fluorescent synthetic compound dipicrylamine (DPA), which serves as a voltage-sensitive FRET acceptor. **b** Cellular resolution voltage imaging with hVOS. Hippocampal slices from hVOS::Fos mouse expressing hVOS probe in granule cells in a Cre-Fos-dependent manner. *Left*: Fluorescence in brain sections after crossing Ai35-hVOS with Cre-Fos mice showing hVOS-expressing neurons in the granule cell layer of the hippocampus. *Right*: Response in four neurons in a hippocampal slice from an hVOS::Fos mouse to electrical stimulation. **c** Schematic representation of VoltageSpy, consisting of the expression of SpyCatcher on the cellular surface and the subsequent extracellular interaction with the VF dye. **d** Subcellular voltage imaging with VoltageSpy. Cultured hippocampal neurons co-expressing SpyCatcher and nuclear mCherry and labeled with VoltageSpy were captured at 500 Hz under widefield fluorescence microscopy. *Left*: VoltageSpy is shown in *green* and nuclear staining in *red*. *Middle*: Higher magnifications of selected dendritic regions. Scale bar 20 μm. *Right*: Voltage imaging in dendrites showing evoked action potentials in selected ROIs, coded by colors indicated in the panel. Images and traces modified with permission from [69] (**b**) and [82] (**d**)

One of the first chemogenetic sensors, named hybrid voltage sensor (hVOS), used an exogenously added lipophilic molecule, which, in a voltage-dependent fashion, quenched fluorescent proteins recruited to the membrane. hVOS employed a two component FRET-based strategy, developed originally without genetic components [68] but adapted to be genetically targetable (Fig. 4a) [69–73]. The first component consists of a fluorescent protein with attached farnesylated and palmitoylated motifs that anchor it to the plasma membrane [70, 72]. The second component is the non-fluorescent synthetic compound dipicrylamine (DPA), which serves as a voltage-sensitive FRET acceptor (quencher). Since DPA is lipophilic but negatively charged, it distributes in the membrane in a voltage-dependent fashion, translocating to the inner layer during depolarization, which quenches protein fluorescence. But since DPA increases membrane capacitance, a low concentration must be used in order not to disturb native physiological responses [73]. Recent use of this sensor shows great versatility to represent neural population activity using cell-specific genetic targeting in transgenic mice (Fig. 4b).

A second type of FRET-based chemogenetic sensors uses microbial rhodopsins as sensors [61, 62]. As mentioned, membrane voltage fluctuations generate a change in the absorption of rhodopsins, which can be read out with a site-specifically ligated organic fluorophore. The fluorophore ligation-assisted rhodopsin electrochromic FRET (FlareFRET) acts as a fluorophore selectively attached to an unnatural amino acid encoded within the rhodopsin [74]. This sensor has wide versatility, allowing addition of a color palette and reaching a 35.9% ΔF/F per 100 mV and millisecond response.

Finally, the recent development of novel rhodamine dyes with high photostability and brightness, such as the Janelia Fluor series (JF), has led to the development of Voltron [42]. JFs are compatible with protein tagging and cross the blood–brain barrier for mammalian in vivo experiments. Voltron combines a voltage-sensitive microbial rhodopsin with a self-labeling protein domain that covalently binds the synthetic JF fluorophore [75, 76]. The voltage-dependent changes in the absorption spectrum of the rhodopsin reversibly modulate the degree of fluorescence quenching of the dye through FRET. With Voltron, one can measure neuronal spiking and subthreshold voltages in larval zebrafish, fruit flies, and mouse brains [42].

#### Enzymatic-based chemogenetic sensors

This design is based on a genetically encoded enzyme on the cell surface, which activates a precursor of an organic voltage indicator. For example, a water-soluble precursor dye is hydrolyzed by an alkaline phosphatase that cleaves off a polar group enhancing its lipophilic character [30]. This greatly improves the targeting and accumulation of the modified electrochromic dye in the membrane of the phosphatase-expressing cell. The aminostyryl-pyridinium (ASP) chromophore is an example of a voltage-sensitive dye precursor with a phosphate group attached to its head group [30, 65]. The first generation of ASP-based dyes resulted in staining of internal organelles within seconds. Using the same strategy, a second generation of sensors using ANNINE-6, one of the most sensitive voltage-sensitive dyes, showed a 50% ΔF/F intensity change per 100 mV and could be used for in vivo targeting [66]. One main advantage of these methods is that membranes can be labeled with large numbers of molecules.

A new generation of enzymatic-based sensor (VF-EX) is a chemogenetic probe in which a genetically encoded esterase uncages a VF dye in defined neurons [77]. VF then uses photoinduced electron transfer (PeT) as a membrane-potential-dependent trigger of fluorescence intensity [78–80]. VF has the speed, brightness, and sensitivity to report action potentials in neurons in single trials. Also, VF is chemically modified to be minimally fluorescent as a precursor and activated upon enzymatic activity. Targeted porcine liver esterase (PLE) on the membrane cleaves VF in the cell surface [81]. Using this approach, action potentials can be measured in cultured neurons [77]. Additionally, compared to some GEVIs [70], VF-EX shows improved SNR and fluorescence change, labeling dendrites and dendritic spines [77].

#### Tag-anchored chemogenetic sensors

A final category of chemogenetic probes traps chemical fluorophores in the plasma membrane by a protein scaffold. The VoltageSpy system employs an engineered cell adhesion molecule interacting with a sarcosine-containing VF dye (Fig. 4a). This interaction is made possible via a polyethyleneglycol (PEG) linker between a small peptide of 13 residues and the VF dye [82]. The localization of VoltageSpy is determined by the expression of the SpyCatcher protein on the cellular surface. Improvement in voltage detection over commonly used genetic voltage indicators in culture cells was reported for VoltageSpy [82]. Using this sensor, one can measure voltages in axon terminals, dendrites, and spines (Fig. 4d). Finally, a hybrid sensor anchored to a protein tag, HAPI-Nile, based on the voltage indicator Nile Red, exhibits fluorescence changes in the physiological range of membrane potential [83]. With this probe, one can detect triggered action potentials and supra/subthreshold activity in cultured neurons.

The selective localization of a synthetic voltage indicator to cells of interest using genetically encoded protein tags seems promising. Some concerns related to these hybrid chemogenetic strategies are their potential toxicity and the selective application of an exogenous lipophilic compound to neuronal membranes in intact tissue for in vivo use.

## Future directions

Voltage imaging, whether with GEVIs, chemogenetics, or other approaches, is amongst the most important challenges in neuroscience. A plethora of new genetically targeted voltage indicators are being designed, to the point that it is difficult to keep up with the literature. In this respect, it would be desirable for a consortium to standardize the haphazard nomenclature of new indicators to enable easier access to this increasingly complex body of work and to prevent confusion (Arch vs ArcLight, or VSD as acronym used for both voltage-sensitive domain and voltage-sensitive dye, for example) or colorful, but not particularly informative, names. It also seems reasonable to establish common standards, calibrations, and benchmarks to test voltage indicators, so one could directly compare their performance under the same conditions, providing accurate measurements of the membrane potential (Box 1; Table 1). Collaborations and integration among different groups working on these difficult problems could benefit the whole field.

**Box 1:** Standards for testing genetic voltage indicators

Direct comparison between different types or families of voltage sensors is difficult in many cases because of a lack of accepted standardized guidelines for the measurement of parameters (see [49] for an attempt). To help developers and users of these tools, we propose a short guideline for standardized testing of genetic voltage indicators, to enable comparing a previously designed voltage sensor with a new one. Although meant for neurons, it could be adapted to other excitable cells. Standard parameters to be reported could include:
(1) Basic measurement parameters, using a light source (halogen lamps, LEDs, one or two photon lasers), power measurements (irradiance, in W/cm^2^) and signal to noise ratio (SNR). Fluorescence changes upon physiological voltage steps, and rise and decay times are also necessary, since they reflect speed and sensitivity of detection. Additionally, optimal absorption/emission spectra for fluorophores and range of light intensity used for testing should be reported.
(2) Photostability is a general benchmark for fluorophores and could determine the temporal range of an experimental design. Measurement of the fluorescence half-life should be included in the same range of light intensities tested.
(3) ∆F/F responses to spontaneous and triggered action potentials (up to 100 Hz) should be reported to predict voltage sensor behavior for measurement of neuronal activity.
(4) As the vast majority of proteins do not have a unique localization in the cellular membrane, providing a detailed description of subcellular localization could help researchers to choose properly and consider any later image analysis for discarding signals from intracellular compartments. For neurons, targeting voltage sensors to somatic, dendritic, or axonal domains is also highly desired and should be highlighted

Current genetically targeted voltage indicators, as well as more traditional organic indicators, appear ready for measurements of neural circuit activity in vitro or in transparent samples in vivo (like *Hydra*, *C. elegans*, *Drosophila* larva, or larval zebrafish, for example). But for use in mammalian or other light-scattering preparations in vivo, new indicators appear necessary. The critical advantage of nonlinear excitation for deep tissue imaging makes it important to design and test indicators specifically for two-photon or, even, for three photon microscopy [84]. A similar case could be made for the design of genetically targeted voltage indicators that exploit second harmonic generation microscopy, a practically unexplored area of genetic engineering (although see [85]). Besides non-linear excitation, other newer imaging methods for in vivo microscopy, such as holographic or temporal multiplexing, adaptive optics (including perhaps index refraction cancelling strategies), computational optics, or Bayesian analysis, also appear necessary to maximize SNR in these challenging imaging conditions with limited photon budgets [86].

Due to the different constraints that need to be met for different types of measurements and experiments, there may not be a single ideal genetic voltage indicator. Thus, custom-designed genetic voltage indicators may have to be designed specifically to measure subthreshold EPSPs or IPSPs (or, alternatively, complementing GEVI with functional dissections of EPSPs or IPSPs), or action potentials, or slow signals. Also, GEVIs with different spectra could enable deeper imaging or optical multiplexing. It seems natural that different genetic voltage indicators should be tailored to specific experiments.

A similar comment about tailoring the method to the experiment can be made about the microscopes: the low photon budget and very fast membrane potential signals are ill-suited for most microscope systems, particularly for those using two-photon excitation. Galvo-based or acousto-optical systems will face major challenges in measuring more than a few cells or points of interest at sufficiently high frequencies. Although there is a new set of spatial and temporal multiplexing techniques, like SLM-based holography [87, 88] to parallelize measurements and make them in 3D [86], they are still far from providing kHz sampling of thousands of neurons in a volume in vivo. The increasing variety of different optical systems provides a rich palette for researchers, who should effectively tailor their microscopy choice to the experiment at hand. Moreover, different combinations of sensors and microscopes could be used to jointly enhance the measurements. In this “hybrid” future, different probes and different optical components might be routinely combined in a single experiment, again emphasizing the need for collaborative ventures.

Finally, it seems that the existing GEVIs and chemogenetic sensors, as exciting as they are, have still only explored a relatively narrow corner of a vast chemical and biological space of possibilities. Essentially all GEVIs belong to two families of voltage-sensitive proteins (phosphatase or rhodopsin-based), whose voltage sensitivities were found serendipitously. Given the importance of voltage imaging for the future of neuroscience, we feel that the field is ripe for a systematic exploration of alternative conceptual design frameworks, exploring in particular the rich molecular and chemical diversity found in biological chromophores. This type of systematic testing and surveying is not ideally suited for individual laboratories, working at the mercy of short-term grant funding, but more appropriately belongs in the realm of large-scale science, either in a national facility (as happens in physics and astronomy) [89] or through the large scale coordination of funding efforts, either through private foundations or national or international BRAIN initiatives [90].
